# Preventive effect of *Dioscorea japonica* on squamous cell carcinoma of mouse skin involving down-regulation of prostaglandin E_2_ synthetic pathway

**DOI:** 10.3164/jcbn.17-54

**Published:** 2018-01-12

**Authors:** Izumi Tsukayama, Keisuke Toda, Yasunori Takeda, Takuto Mega, Mitsuki Tanaka, Yuki Kawakami, Yoshitaka Takahashi, Masumi Kimoto, Kei Yamamoto, Yoshimi Miki, Makoto Murakami, Toshiko Suzuki-Yamamoto

**Affiliations:** 1 Department of Nutritional Science, Okayama Prefectural University, 111 Kuboki, Soja, Okayama 719-1197, Japan; 2 Lipid Metabolism Project, Tokyo Metropolitan Institute of Medical Science, Setagaya-ku, Tokyo 156-8506, Japan; 3 Graduate School of Technology, Industrial and Social Science, Tokushima University, Minami-jyosanjima, Tokushima 770-8513, Japan; 4 PRIME, Japan Agency for Medical Research and Development, Chiyoda-ku, Tokyo 100-0004, Japan; 5 Laboratory of Microenvironmental and Metabolic Health Sciences, Center for Disease Biology and Integrative Medicine, Faculty of Medicine, The University of Tokyo, 7-3-1 Hongo, Bunkyo-ku, Tokyo 113-8655, Japan; 6 AMED-CREST, Japan Agency for Medical Research and Development, Chiyoda-ku, Tokyo 100-0004, Japan

**Keywords:** wild yam, inflammation, carcinogenesis, skin cancer, prostaglandin E_2_

## Abstract

Hyperproduced prostaglandin E_2_ by cyclooxygenase-2 and microsomal prostaglandin E synthase-1 evokes several pathophysiological responses such as inflammation and carcinogenesis. Our recent study demonstrated that *Dioscorea japonica* extract suppressed the expression of cyclooxygenase-2 and microsomal prostaglandin E synthase-1 and induced apoptosis in lung carcinoma A549 cells. In the present study, we investigated the effects of *Dioscorea japonica* on squamous cell carcinoma of mouse skin. *Dioscorea japonica* feeding and *Dioscorea japonica* extract topical application suppressed the expression of cyclooxygenase-2, microsomal prostaglandin E synthase-1, interleukin-1β and interleukin-6 and inhibited tumor formation, hyperplasia and inflammatory cell infiltration. Immunohistochemical analyses showed the immunoreactivities of cyclooxygenase-2 and microsomal prostaglandin E synthase-1 in tumor keratinocytes and stronger immunoreactivities of cyclooxygenase-2 and hematopoietic prostaglandin D synthase in epidermal dendritic cells (Langerhans cells). Treatment with *Dioscorea japonica* decreased the immunoreactivity of cyclooxygenase-2 and microsomal prostaglandin E synthase-1. These results indicate that *Dioscorea japonica* may have inhibitory effects on inflammation and carcinogenesis via suppression of the prostaglandin E_2_ synthetic pathway.

## Introduction

Prostaglandin (PG) E_2_, a lipid mediator, is derived from arachidonic acid, and is involved in several pathophysiological responses such as inflammation, inhibition of gastric acid secretion, pain transmission, neurodegeneration and carcinogenesis.^(1)^ In the PGE_2_ synthetic pathway, the isozymes cyclooxygenase (COX)-2 and microsomal PGE synthase-1 (mPGES-1) are induced by the pathophysiological conditions, and the hyperproduced PGE_2_ evokes the disease.^(2,3)^ Clinically, COX inhibitors, namely non-steroidal anti-inflammatory drugs (NSAIDs), are commonly used for pyretolysis and pain relief,^(2)^ however, most NSAIDs target constitutive COX-1 and inducible COX-2 and cause side effects such as gastrointestinal toxicity and cardiovascular risk.^(4)^ On the other hand, a few mPGES-1 inhibitors have been found,^(3)^ and none have been used clinically. Thus, it would be beneficial to identify natural substances from foods that have safe and functional effects on the regulation of PGE_2_ production.

*Dioscorea japonica*, a wild yam, is a relative of the *Dioscoreaceae* family native to Japan. *Dioscoreaceae* yam tubers are usually edible and are rich in many nutrients.^(5,6)^ *Dioscorea japonica *is good for nutritional fortification,^(7–10)^ and it has a gastric mucosal protective effect conferred by glycoproteins and digestive enhancement by glycosidase. Recently, we found that *Dioscorea japonica* extract (DJE) suppressed the expression of COX-2 and mPGES-1 in human non-small-cell lung carcinoma A549 cells and colon carcinoma Caco-2 cells, thereby inducing apoptosis in such cells.^(11)^ DJE suppresses COX-2 mRNA with translocation of transcriptional factor nuclear factor-κB (NF-κB) to cytosol and a reduction of COX-2 promoter activity.^(11)^

In this study, to confirm the effects of *Dioscorea japonica* on inflammation and carcinogenesis via the suppression of COX-2 and mPGES-1 *in vivo* we demonstrate a two-stage cutaneous chemical carcinogenesis model of mouse skin.^(12)^

## Materials and Methods

### Animal rearing conditions and the method of preparing a model of squamous cell carcinoma

**Fig. 1:**
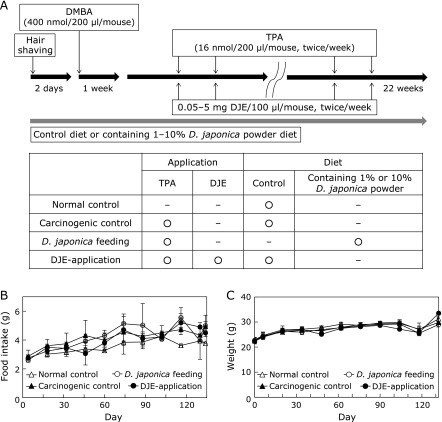
Treatment scheme and comparison of food intake and body weight among the experimental groups. Seven-week-old male Balb/c mice were treated with the respective regimens according to the treatment scheme (A). Animals were divided into four experimental groups: group “normal control” was given control diet and treatment with vehicle; group “carcinogenic control” was given normal diet and treatment with DMBA/TPA; group “*Dioscorea japonica* feeding” (*D. japonica* feeding) was given 1% or 10% *Dioscorea japonic*a powder (w/w) containing diet and treatment with DMBA/TPA; group “*Dioscorea japonica* extract topical application” (DJE-application) was given a normal diet and treatment with DMBA/TPA and 0.05–5 mg of *Dioscorea japonica* extract/100 µl of 50% ethanol. Food intake (B) and body weight (C) were recorded. The values represent mean ± SD.

All protocols were approved by the Exclusive Committee on Animal Research at Okayama Prefectural University and the research was conducted in conformity with the Public Health Service (PHS) policy. Seven-week-old male Balb/c mice had free access to drinking water and food. Mice were fed a powder diet (CRF-1; Oriental Yeast Co., Ltd., Tokyo, Japan) with or without (w/w) 1% or 10% *Dioscorea japonica* powder. *Dioscorea japonica* was obtained from Autoraimu Yoshio Ltd. (Niimi, Japan). The outer skin of *Dioscorea japonica* was pared away, and it was dried and pulverized at 40°C. Feed consumption was measured twice a week, and weight was measured every second week. According to the procedure of Modi *et al.*,^(12)^ a squamous cell carcinoma model was induced by topically applying the following to each mouse: firstly, 200 µl of 2 mM 7,12-dimethylbenz[*a*]anthracene (DMBA, as an initiator, Sigma-Aldrich, St. Louis, MO); then, after one week, 200 µl of 80 µM 12-*O*-tetradecanoylphorbol-13 acetate (TPA, as a promoter, Sigma-Aldrich) twice per week for 22 weeks (Fig. 1A). Numbers and volumes of cutaneous papillomas were measured. Mice in three experimental groups were applied with 100 µl of 0.05, 0.5 or 5 mg of *Dioscorea japonica* extract eluted from the powder with 50% ethanol 30 min before the application of TPA, and fed a basal diet (CRF-1) without *Dioscorea japonica* powder. 

### Quantitative reverse transcriptase (RT)-PCR

The gene expression was analyzed by quantitative RT-PCR (iQ5 real-time PCR system, Bio-Rad, Hercules, CA) using cDNA prepared from isolated total RNA. The quantitative PCR was performed using SsoAdvanced Universal SYBR Green Supermix (Bio-Rad), and the following PCR primer pairs: *Ptgs1* (COX-1), 5'-CTTTGC ACAACACTTCACCCACC-3' (forward) and 5'-AGCAACCCA AACACCTCCTGG-3' (reverse); *Ptgs2* (COX-2), 5'-GCATTC TTTGCCCAGCACTT-3' (forward) and 5'-AGACCAGGCACC AGACCAAAGA-3' (reverse); *Ptges* (mPGES-1), 5'-CTGCTG GTCATCAAGATGTACG-3' (forward) and 5'-CCCAGGTAG GCCACGGTGTGT-3' (reverse); *Ptges2* [membrane-associated PGES-2 (mPGES-2)], 5'-AAGACATGTCCCTTCTGC-3' (forward) and 5'-CCAAGATGGGCACTTTCC-3' (reverse); *Ptges3* [cytosolic PGES (cPGES)], 5'-AGTCATGGCCTAGGTTAAC-3' (forward) and 5'-TGTGAATCATCATCTGCTCC-3' (reverse); *Ptgds2* [hematopoietic PGD synthase (H-PGDS)], 5'-CACGCTGGAT GACTTCATGT-3' (forward) and 5'-AATTCATTGAACATCCG CTCTT-3' (reverse); *Il1b* [interleukin (IL)-1β], 5'-GTCACAAGA AACCATGGCACAT-3' (forward) and 5'-GCCCATCAGAGG CAAGGA-3' (reverse); *Il6* (IL-6), 5'-CTGCAAGAGACTTCC ATCCAGTT-3' (forward) and 5'-AGGGAAGGCCGTGGTTGT-3' (reverse); and *gapdh* [glyceraldehyde 3-phosphate dehydrogenase (GAPDH)], 5'-TGAACGGGAAGCTCACTGG-3' (forward) and 5'-TCCACCACCCTGTTGCTGTA-3' (reverse). The relative expression levels were shown against normal controls and represent mean ± SD.

### Pathohistological and immunohistochemical analyses

Prepared paraffin-embedded sections of mouse skin were used for hematoxylin and eosin (HE) staining and immunohistochemical staining. For single immunolabeling, sections were treated with 3% hydrogen peroxide to block endogenous peroxidase activity, and were blocked with 12.5% Block Ace (DS Pharma Biomedical Co., Ltd., Tokyo, Japan) to block nonspecific bindings. For immunolabeling, we used the following first antibodies: goat anti-COX-2 antibody (1:50, catalogue no. sc-1745, lot no. E102, Santa Cruz Biotechnology, Inc., Santa Cruz, CA), rabbit anti-mPGES-1 antibody (1:500, catalogue no. 160140, lot no. 0436398-1, Cayman Chemical Co., Ann Arbor, MI), rabbit anti-H-PGDS antiserum (1:1,000, catalogue no. 10004348, lot no. 0451181-1, Cayman Chemical Co.), rat anti-Ly-6G/Ly-6C (Gr-1) antibody (1:500, a marker of neutrophils; catalogue no. 108413, lot no. B141536, BioLegend, San Diego, CA), and mouse anti-Langerin/CD207 antibody (1:1000, a marker of Langerhans cells; catalogue no. DDX0361P-50, lot no. DDX0361-009, DENDRITICS, Lyon, France), and the following second antibodies: biotinylated anti-goat, biotinylated anti-rabbit, Cy3-labeled donkey anti-goat, FITC-labeled donkey anti-rabbit, FITC-labeled donkey anti-goat, FITC-labeled donkey anti-rat, FITC-labeled donkey anti-mouse IgGs (1:400, biotinylated IgGs from Vector Laboratories, Burlingame, CA, and fluorescein-labeled IgGs from Jackson ImmunoResearch Laboratories, Inc., West Grove, PA). The biotin-labeling was enhanced by ABC Elite kit (Vector Laboratories) and was visualized in 50 mM Tris (pH 7.6) containing 0.1% 3,3'-diaminobenzidine tetrahydrochloride (DAB) and 0.01% hydrogen peroxide at 37°C. The fluorescein-labeled sections were examined by confocal laser scanning light microscopy (CLSM, Fluoview FV1000, Olympus Co., Tokyo, Japan).

### Electrospray ionization mass spectrometry (ESI-MS)

All procedures were performed as described previously.^(13)^ Tissues were homogenized with a Polytron homogenizer in methanol and were incubated overnight. As internal standards for determination, 100 pmol of *d5*-labeled eicosapentaenoic acid (EPA) and *d8*-labeled 15-hydroxyeicosatetraenoic acid (15-HETE), were added to the samples. The lipids in the sample were extracted using Oasis HLB cartridges (Waters, Milford, MA), and were dried up under nitrogen gas. The analysis was performed using a 4000Q-TRAP quadrupole-linear ion trap hybrid mass spectrometer (AB Sciex, Framingham, MA) with LC (NexeraX2 system; Shimadzu Co., Kyoto, Japan). The sample was applied to a C18 column (Kinetex C18, 2.1 × 150 mm, 1.7 µm, Phenomenex, Inc., Torrance, CA) coupled for ESI-MS/MS. For analyses of fatty acids, the samples are applied to a column and separated by a step gradient with mobile phase A (acetonitrile:MeOH:water = 1:1:1 (v/v/v) containing 5 µM phosphoric acid and 1 mM ammonium formate) and mobile phase B (2-propanol containing 5 µM phosphoric acid and 1 mM ammonium formate) at a flow rate of 0.2 ml/min at 50°C. Whereas, for analyses of oxidized fatty acids, the samples are applied to a column and separated by a step gradient with mobile phase C (water containing 0.1% acetic acid) and mobile phase D (acetonitrile:MeOH = 4:1; v/v) at a flow rate of 0.2 ml/min at 45°C. Signature ion fragments for each targeted lipid were monitored and quantified by a multiple reaction monitoring (MRM) method. Identification was conducted using MRM transition^(14)^ and retention times. Quantification was performed based on peak area of the MRM transition and the calibration curve obtained with authentic standard for each compound.

### Effect of diosgenin on expression of COX-2 and mPGES-1 in RAW264 cells

Mouse macrophage-like RAW264 cells was obtained from RIKEN BioResource Center (Tsukuba, Japan). The cells were cultured in Dulbecco’s modified Eagle’s medium supplemented with 10% fetal bovine serum, 100 U/ml penicillin, and 100 µg/ml streptomycin. RAW264 cells were incubated with or without 100 nM diosgenin. After the incubation for 3 h, the cells were stimulated by 2.5 µg/ml lipopolysaccharide (LPS) for 6 h. mRNA expression of *Ptgs2* and *Ptges* was measured by quantitative RT-PCR.

### Statistics

Data were statistically evaluated by ANOVA with Bonferroni’s or Dunnett’s post-hoc test at significance level of *p*<0.01 or *p*<0.05.

## Results

### Comparison of tumor formation among the experimental groups

**Fig. 2:**
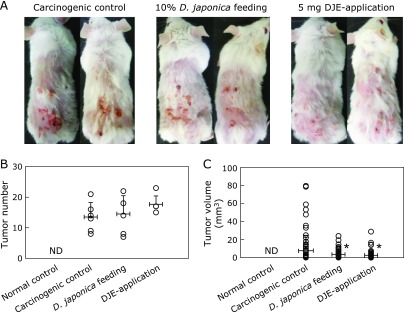
Comparison of tumor formation among experimental groups. Representative images of mice from each experimental group after 22 weeks of tumor induction are shown in (A). Tumors are identified to enable comparison between normal skin and swelling tissue, and tumor number (B) and volume (C) are indicated. The values represent mean ± SD of 6 mice per group; *p<0.01 compared with the carcinogenic control.

Figure 2 shows tumor formation in the experimental groups including *Dioscorea japonica* feeding group and DJE topical application group. Throughout the experiment, food intake and body weight were not significant differences among the experimental groups (Fig. 1B and C). After 22 weeks of tumor induction, there was no noticeable difference in tumor numbers between the experimental groups with the exception of the normal control (Fig. 2B and Supplemental Fig. 1A*). However, the tumor volumes were significantly decreased in the *Dioscorea japonica* feeding and DJE topical application groups (Fig. 2A, C and Supplemental Fig. 1B*). These data indicate that *Dioscorea japonica* treatment is more beneficial for tumor volumes than tumor numbers. Thus, *Dioscorea japonica* may inhibit tumor growth.

### Suppression of COX-2, mPGES-1, inflammatory cytokines, and decrease of PG products by *Dioscorea japonica* treatment

**Fig. 3:**
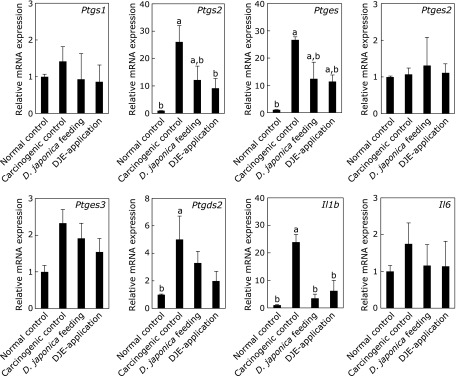
Changes in gene expression. In normal or carcinogenic controls, 10% *Dioscorea japnonica* feeding, and 5 mg DJE topical application, mRNA expression of *Ptgs1* (COX-1), *Ptgs2* (COX-2), *Ptges* (mPGES-1), *Ptges2* (mPGES-2), *Ptges3* (cPGES), *Ptgds2* (H-PGDS), *Il1b* (IL-1β), and *Il6* (IL-6) was analyzed by quantitative RT-PCR. Expression levels of each mRNA were normalized to that of *gapdh* (GAPDH) mRNA. The relative expression levels are shown against normal control levels and represent mean ± SD of 6 mice per group; *p*<0.01 compared with ^a^normal controls and ^b^carcinogenic controls.

**Fig. 4:**
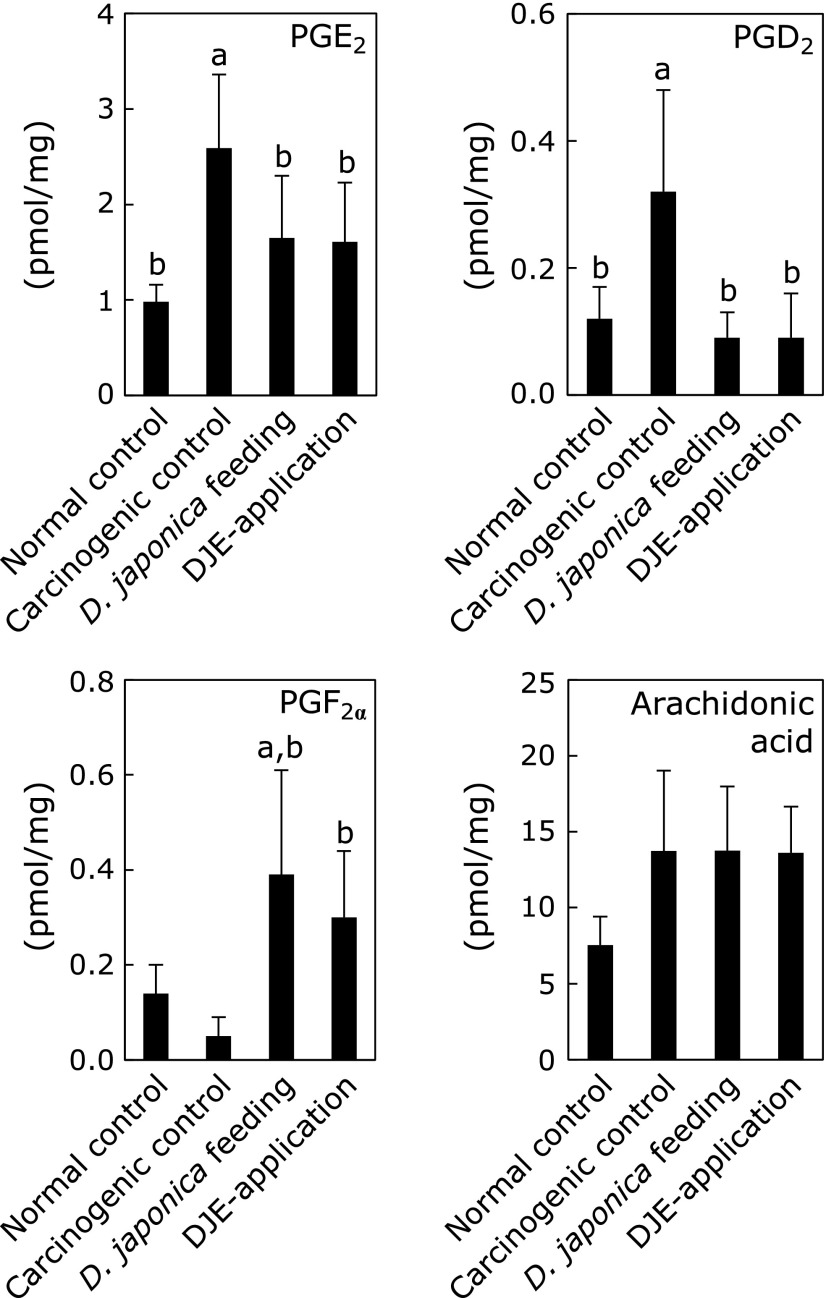
Changes in amounts of PGs. The production of PGE_2_, PGD_2_, PGF_2__α_ and arachidonic acid was analyzed by ESI-MS. Amounts indicated represent mean ± SD of 10 samples per group; *p*<0.01 compared with ^a^normal controls and ^b^carcinogenic controls.

To examine the effect of *Dioscorea japonica* administration on mRNA expression in mouse skin (Fig. 3), *Ptgs1* (COX-1), *Ptgs2* (COX-2), *Ptges* (mPGES-1), *Ptges2* (mPGES-2), *Ptges3* (another cytosolic PGE_2_ synthase, cPGES), *Ptgds2* (PGD_2_ synthesizing enzyme, H-PGDS), and inflammatory cytokines *Il1b* (IL-1β) and *Il6* (IL-6) were analyzed by quantitative RT-PCR. In the carcinogenic control mice, *Ptgs2*, *Ptges* and *Il1b* increased approximately 25-fold compared with the normal control mice. On the other hand, *Dioscorea japonica* feeding led to the suppression of *Ptgs2*, *Ptges* and *Il1b* mRNAs to 47%, 46% and 15% compared with the carcinogenic control, respectively. In addition, DJE topical application decreased mRNA levels of these genes to 35%, 43% and 26% compared with the carcinogenic control, respectively. Moreover, mRNA expression of *Il6* in *Dioscorea japonica* treatment was also decreased to almost the same level to that in normal control. Additionally, *Dioscorea japonica* treatment did not affect to the expression of *Ptgds2* and *Ptges3*, although the expression of them was induced to approximately 5-fold and 2.5-fold, respectively in the carcinogenic control mice (Fig. 3). On the other hand, the expression of *Ptgs1* and *Ptges2* was not affected among all experimental groups (Fig. 3). Furthermore, lipid metabolome analysis in mouse skin was performed by liquid chromatography tandem mass spectrometry (ESI-MS, Fig. 4). We confirmed that the main lipid mediator in mouse skin was PGE_2_, and the production of PGE_2_ and PGD_2_ in *Dioscorea japonica *feeding mice decreased to 63.7% and 28.1%, respectively, compared with the carcinogenic control. DJE topical application also decreased PGE_2_ and PGD_2_ production to 62.2% and 28.1%, respectively. Whereas, PGF_2__α_ increased in *Dioscorea japonica* treatment groups.

### Effect of *Dioscorea japonica* treatment on pathohistological and immunohistochemical analyses

**Fig. 5:**
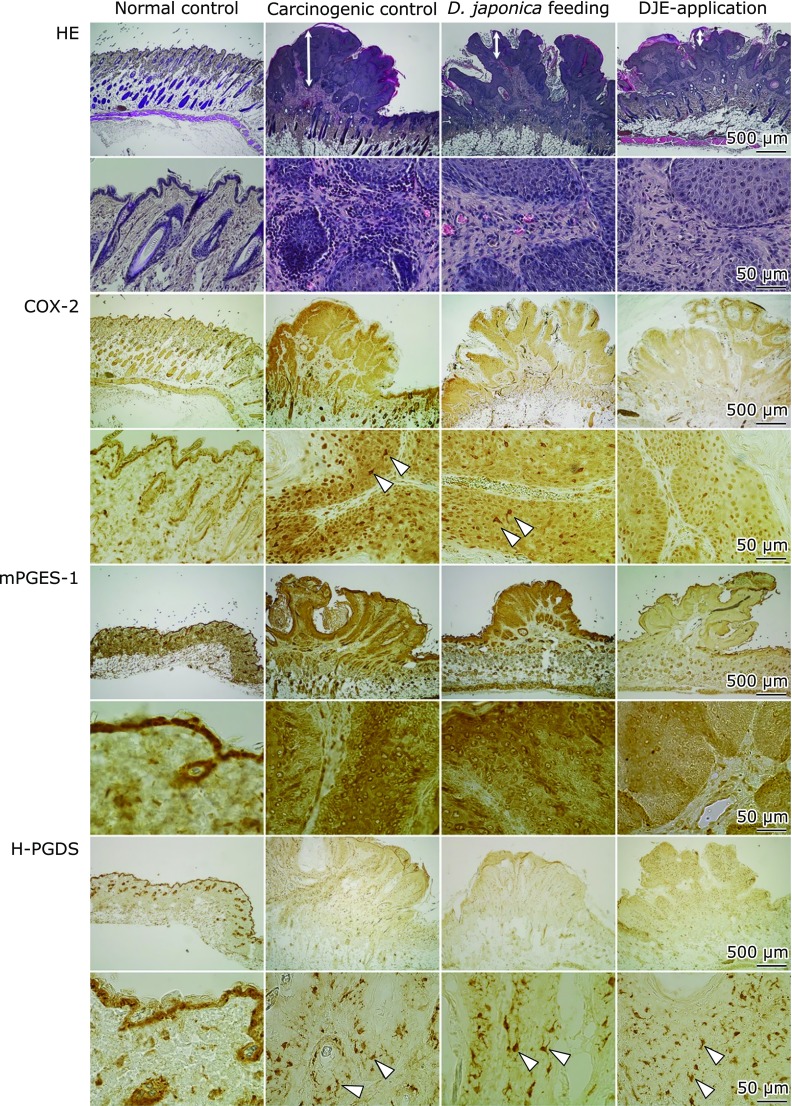
Histochemical analyses in mouse skin. Hematoxylin and eosin (HE) staining were pathologically analyzed. Brown-colored immunostaining of COX-2, mPGES-1 and H-PGES indicates each localization. Double-headed arrows indicate hyperplasia of accumulated cancer cells and arrowheads indicate Langerhans cells.

**Fig. 6:**
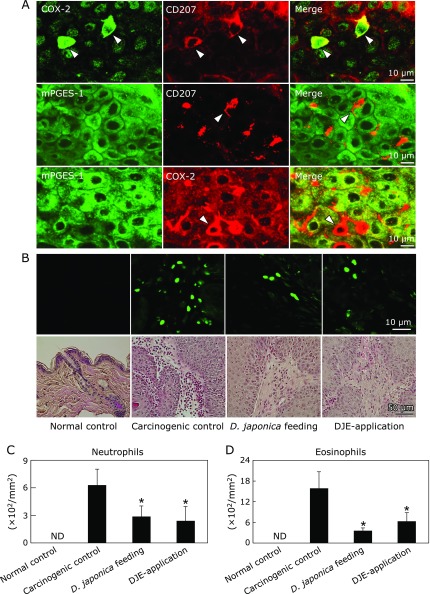
Double fluorostaining of COX-2, mPGES-1 or CD207 (A), and measurement of infiltrating cells in mouse skin (B–D). COX-2 and mPGES-1 expressing cells in carcinogenic control mouse epidermis were analyzed by immunofluorescent staining (A). COX-2 and CD207 (marker of Langerhans cells) were co-fluorostained, but mPGES-1 and CD207 were not. mPGES-1 and COX-2 were co-fluorostained in carcinogenic keratinocytes. For double immunofluorescent staining, merged signals are shown in yellow. The number of infiltrating neutrophils was counted in visualized by immunofluorescent staining of Ly-6G/Ly-6C (Gr-1) (green in upper B) and eosinophils were counted after HE staining (bottom B). The comparison of their number was represented as mean ± SD of 10 sections per group (C and D); *p<0.01 compared with the carcinogenic control. Arrowheads indicate Langerhans cells. ND, not detected.

 Carcinogenic control mouse epidermis induced exhibited significant epidermal hyperplasia with hyperproliferation of the keratinocytes (upper part of HE staining in Fig. 5). *Dioscorea japonica* treatment markedly suppressed the epidermal hyperplasia. Moreover, infiltration of a lot of inflammatory cells in the epidermis in carcinogenic control was substantially inhibited by *Dioscorea japonica* feeding and DJE topical application (lower part of HE staining in Fig. 5). Immunohistochemical analyses indicated that COX-2 and mPGES-1 localized in tumor keratinocytes, and, additionally, COX-2 was strongly expressed in epidermal dendritic cells (Langerhans cells, LCs), but mPGES-1 was not observed in LCs (Fig. 5). Similarly, H-PGDS was also highly expressed in LCs, but not in keratinocytes. Interestingly, COX-2 staining was decreased in both tumor keratinocytes and LCs by *Dioscorea japonica* treatment. Additionally, we determined the localization of COX-2 and mPGES-1 in the mouse epidermis with inflammation and tumorigenesis by double immunofluorescent staining. Consistent with immunohistochemical analyses, COX-2 co-localized with CD207 (LC marker) and was present both in cancer cells and LCs. The immunoreaction of COX-2, especially in LCs, was stronger than that in cancer cells (Fig. 5 and 6A). In contrast, double staining for mPGES-1 and CD207, or for mPGES-1 and COX-2 showed that mPGES-1 co-localized with COX-2 in cancer cells, but not in LCs (Fig. 6A).

Furthermore, we conducted immunostaining and HE staining, and counted the number of neutrophils and eosinophils to determine the effect of *Dioscorea japonica* on inflammatory cell infiltration. As shown in Fig. 6B, the greatest numbers of neutrophils and eosinophils were observed in carcinogenic controls. Counting the cells showed that *Dioscorea japonica *treatment decreased the infiltrating perilesional neutrophils and eosinophils to less than 50% and 20–35%, respectively (Fig. 6C and D).

## Discussion

COX-2 is functionally coupled with mPGES-1 for PGE_2_ synthesis in pathophysiological conditions,^(15)^ and these enzymes are overexpressed in various tumors and inflammation.^(2,15–17)^ In skin cancers caused by some stimuli such as ultraviolet light and chemicals, COX-2 is induced and PGE_2_ is increased.^(18,19)^ While COX-2 overexpression certainly induces skin carcinogenesis,^(20)^ studies using COX-2 inhibitor^(21)^ and COX-2 deficient mouse^(22)^ demonstrate the prevention of tumor development in squamous cell carcinoma of the mouse skin. Our previous study demonstrated for the first time that DJE suppressed the expression of COX-2 and mPGES-1, causing PGE_2_ decrease and cancer cell apoptosis in lung cancer A549 cells.^(11)^ In the present study, we confirmed the novel functionality of *Dioscorea japonica* that inhibited cancer evolution via suppression of COX-2 and mPGES-1, *in vivo* using a model of squamous cell carcinoma of the skin.

In the present model of squamous cell carcinoma, DMBA as an initiator is incorporated into LCs, and is metabolized to DMBA-3,4-diol-1,2-epoxide (DMBADE).^(12)^ DMBADE is known to strongly induce keratinocytes during carcinogenesis.^(12,23)^ We also showed that not only COX-2 but also mPGES-1 was overexpressed in squamous cell carcinoma of mouse skin, and they were co-localized in proliferated epidermal cancer cells. In addition, COX-2 was highly expressed, but mPGES-1 was not present in LCs. According to our results, COX-2 may be involved in carcinogenesis, tumor growth, and activation of LCs. On the other hand, main roles of mPGES-1 may be proliferation and development of cancer cells. PGE_2_ specific receptors EP1-4 are expressed in normal and tumor epidermis.^(24,25)^ In tumors induced by UVB light exposure and COX-2 overexpression, EPs localize in epidermal hyperplasia.^(24,25)^ The signaling pathways of PGE_2_/EP1 and PGE_2_/EP3 are involved in immune responses of the skin via T helper type (Th) 1 cells and dendritic cells, respectively.^(26,27)^ Although the presence of EP4 in LCs is immunohistochemically unknown, PGE_2_/EP4 signaling promotes migration and maturation of Langerhance cells, and initiates skin immune responses.^(28)^ Taken together with our results, it is suggested that the produced PGE_2_ by COX-2 and mPGES-1 induce tumor development and immune responses via the individual receptors in the autocrine or paracrine manner in squamous cell carcinoma of the skin.

In addition, H-PGDS was also predominantly expressed in LCs, and which may indicate a coupling of COX-2 and H-PGDS and the produced PGD_2_ regulates LCs activity. LCs play a key role in establishment of cutaneous immunity, and participate in squamous cell carcinoma.^(12,29)^ Human dendritic cells including LCs in the skin express H-PGDS, and produce PGD_2_ in response to various stimuli.^(30)^ Thus it is suggested that the produced PGD_2_ in autocrine manner in LCs is involved in cutaneous immune system and inflammatory reactions in the skin. Previous reports indicate the opposing immunomodulatory roles of PGD_2_ receptors, DP and CRTH2 (chemoattractant receptor homologous molecule expressed on Th2 cells) .^(31,32)^ In inflammatory processes of the skin, PGD_2_ may play a role in the early stages via DP, and in eosinophil migration during the late stages via CRTH2. In each process of developing squamous cell carcinoma, further studies will be needed to determine the roles of H-PGDS and PGD_2_ in inflammation and carcinogenesis.

**Fig. 7:**
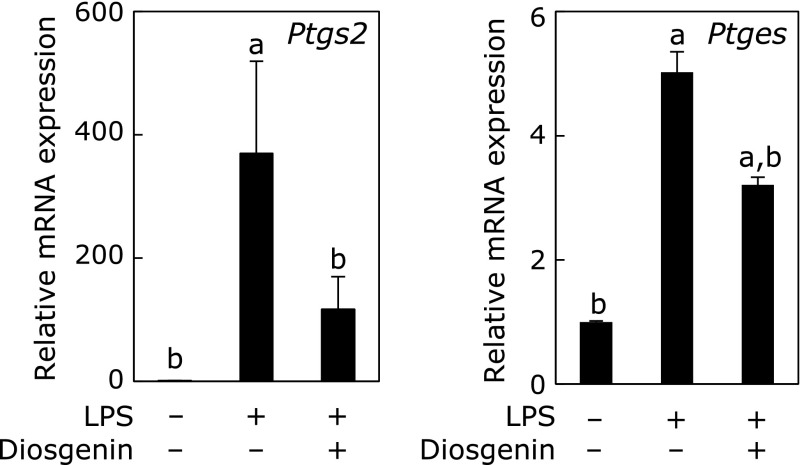
Effects of diosgenin on the expression of *Ptgs2* and *Ptges* in LPS-stimulated RAW264 cells. LPS-stimulated RAW264 were cultured with or without 100 nM diosgenin. mRNA expression of *Ptgs2* (COX-2) and *Ptges* (mPGES-1) was measured by quantitative RT-PCR. The expression levels are shown as a relative value against control cells without LPS and diosgenin, and are represented mean ± SD of 3 separate experiments; *p*<0.05 compared with ^a^LPS(–)/diosgenin(–) and ^b^LPS(+)/diosgenin(–).

Our recent report demonstrates that *Dioscorea japonica* suppresses the expression of COX-2 and mPGES-1, and has an anti-carcinogenic effect on several model cell lines (reference 11 and unpublished data). In the present study, not only application, but also ingestion of *Dioscorea japonica* affects the expression of COX-2 and mPGES-1, and leads to decreased PGE_2_ *in vivo*. Previous studies concerning phytochemical effects on the PGE_2_ synthetic pathway report the suppression of COX-2 by resveratrol,^(33)^ humulon,^(34)^ chrysin,^(35)^ and 6-shogaol^(36)^ and of mPGES-1 by sulforaphane^(37)^ and curcumin.^(38)^ Moreover, we demonstrated that *Dioscorea japonica* was effective in suppressing both COX-2 and mPGES-1 mRNAs. *Dioscorea japonica* is rich in a lot of nutrients, and one of them is a plant steroidal saponin such as diosgenin. Diosgenin has been reported to have some preventive effects on mouse colon carcinogenesis,^(39)^ mouse squamous cell carcinoma^(40)^ and mouse Alzheimer’s disease.^(41)^ Our previous study also showed that diosgenin suppressed COX-2 in A549 cells,^(11)^ and additionally our recent experiment showed that it suppressed both of COX-2 and mPGES-1 in LPS-stimulated mouse macrophage-like RAW264 cells (Fig. 7). Therefore, diosgenin is likely one of the effective substances in the present study, and liposoluble and low molecular diosgenin may have effects via the oral and application routes.

In concluding, our *ex vivo*^(11)^ and *in vivo* (present study) results suggest that *Dioscorea japonica* may have inhibitory effects on inflammation and carcinogenesis caused by the hyperexpression of COX-2 and mPGES-1. In the present study, *Dioscorea japonica* also exerts a preventive effect on squamous cell carcinoma as a refractory skin disease, which has few known specific agents and treatments. Our results on the effects of *Dioscorea japonica* may pave the way for further therapeutic methods.

## Abbreviations

COX: cyclooxygenase
cPGES: cytosolic prostaglandin E synthase
CRTH2: chemoattractant receptor homologous molecule expressed on Th2
DJE: *Dioscorea japonica* extract
DMBA: 7,12-dimethylbenz[*a*]anthracene
DMBADE: 7,12-dimethylbenz[*a*]anthracene-3,4-diol-1,2-epoxide
EPA: eicosapentaenoic acid
GAPDH: glyceraldehyde 3-phosphate dehydrogenase
HE: hematoxylin and eosin
HETE: hydroxyeicosatetraenoic acid
H-PGDS: hematopoietic prostaglandin D synthase
IL: interleukin
LCs: Langerhans cells
LPS: lipopolysaccharide
mPGES-1: microsomal prostaglandin E synthase-1
mPGES-2: membrane-associated prostaglandin E synthase-2
MRM: multiple reaction monitoring
NF-κB: nuclear factor-κB
NSAIDs: non-steroidal anti-inflammatory drugs
PG: prostaglandin
TPA: 12-*O*-tetradecanoylphorbol-13 acetate
