# Supplementary material for: Preventive effect of Dioscorea japonica on squamous cell carcinoma of mouse skin involving down-regulation of prostaglandin E2 synthetic pathway
Source: J Clin Biochem Nutr. 2018 Jan 12;62(2):139–47. doi: 10.3164/jcbn.17-54 (PMC5874233; doi:10.3164/jcbn.17-54)
Supplement: Supplemental Fig. 1 [file jcbn17-54sf01.pdf]

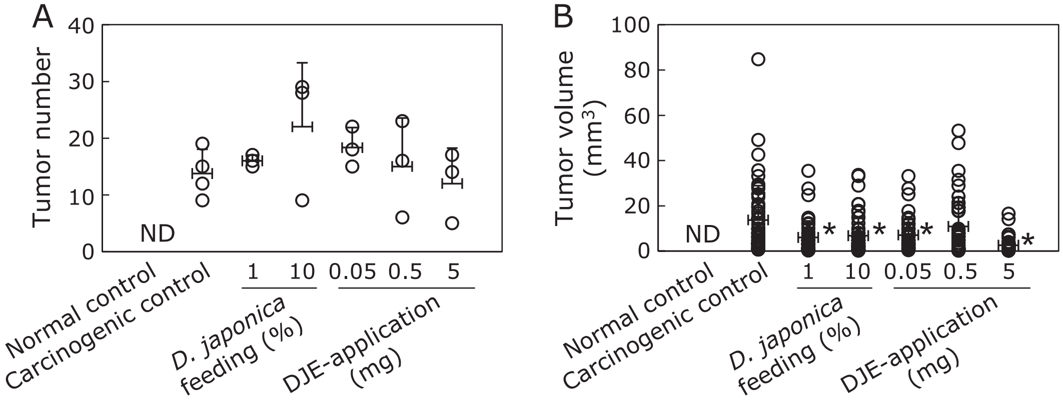

Supplemental Fig. 1. Dose-dependent effects of *D. japonica* feeding and DJE-application on tumor formation. Tumor number (A) and volume (B) were measured. The values represent mean ± SD of 3–4 mice per group; **p*<0.01 compared with the carcinogenic control. ND; not detected.
